# A48 LYOPHILIZED FECAL MICROBIOTA TRANSPLANT (FMT) DELAYS THE ONSET OF SPONTANEOUS COLITIS IN MUC2-/- MICE

**DOI:** 10.1093/jcag/gwac036.048

**Published:** 2023-03-07

**Authors:** J J Chan, C Chan, H P Sham, B Vallance

**Affiliations:** 1 Pediatrics, University of British Columbia; 2 Gut; 3 Health, BC Children's Hospital Research Institute, Vancouver, Canada

## Abstract

**Background:**

Fecal microbiota transplant (FMT) is the transfer of fecal microbes from a healthy donor to a recipient to normalize gut microbiota. FMT therapy has been effectively used to prevent recurrent *Clostridium difficile* infections. While there are emerging studies applying FMT to other gastrointestinal (GI) disorders including inflammatory bowel disease (IBD), its efficacy in treating IBD, and the mechanisms involved remain unclear. In the GI tract, the mucus barrier plays a critical role in protecting the underlying epithelium against harmful luminal stimuli. The secreted mucin 2 (MUC2) is a glycosylated protein and the major component of the GI mucus layer. *Muc2* deficient (^-/-^) mice are used to model IBD because they develop a leaky gut as well as spontaneous colitis.

**Purpose:**

We investigated the efficacy of FMT in attenuating the development of spontaneous colitis in *Muc2*^-/-^ mice. This study also evaluated whether FMT using lyophilized stool can successfully alter the gut microbiome of recipient mice.

**Method:**

To achieve successful colonization of transplanted microbiota, *Muc2*^-/-^ mice were pretreated prior to FMT with the antifungal amphotericin B, followed by an antibiotic mixture of metronidazole, ampicillin, neomycin and vancomycin in their drinking water for ten days. For two consecutive days, FMT was administered to *Muc2*^-/-^ mice via consumption of lyophilized stool cakes created from donor C57BL/6 mice, while sucrose cakes were given as control. The fecal microbiome was analyzed at baseline, post-antibiotics and biweekly post-FMT using 16S rRNA sequencing. We monitored body weight and signs of spontaneous colitis, including rectal prolapse, until the mice reached their humane endpoint or turned 30 weeks old.

**Result(s):**

Following antibiotic pretreatment, *Muc2*^-/-^ mice experienced significant weight loss, with 15-20% requiring euthanization within ten days of receiving antibiotics. Unless FMT was administered post-antibiotics, 95% of the mice developed rectal prolapse or otherwise reached their humane endpoint. In comparison, FMT-treated mice rarely developed rectal prolapse, with 55-80% of the FMT-treated mice surviving until the end of the experiment; some FMT-treated mice had no signs of rectal prolapse when euthanized at 30 weeks. Therefore, FMT significantly improved the survival rate of antibiotic-treated *Muc2*^-/-^ mice and delayed the onset of rectal prolapse / spontaneous colitis. Microbiome analysis revealed that the lyophilized FMT altered the fecal microbiome as compared to mice receiving the sucrose control.

**Conclusion(s):**

Notably, FMT increased the survival rates of antibiotic-pretreated *Muc2*^-/-^ mice while delaying the onset of rectal prolapse. This indicates that lyophilized FMT counteracts the typical spontaneous colitis and weight loss seen in *Muc2*^-/-^ mice. Our findings can offer clinically relevant insight into how FMT induces a shift in the gut microbiome and its potential usage in treating IBD, as well as other gastrointestinal conditions.

**Disclosure of Interest:**

None Declared

